# Effects of Norbixin on Growth Performance, Immunomodulation, Antioxidant Systems, and Metabolic Alterations in the Oriental River Prawn (*Macrobrachium nipponense*): An Integrative Analysis

**DOI:** 10.1002/vms3.71014

**Published:** 2026-06-15

**Authors:** Mohammad Ettefaghdoost, Hossein Haghighi, Adeleh Haghdoost

**Affiliations:** ^1^ Fisheries Department Faculty of Natural Resources University of Guilan Sowmeh Sara, Guilan Iran; ^2^ Department of Animal Science Faculty of Agriculture Ferdowsi University of Mashhad Mashhad Iran

**Keywords:** antioxidants, growth, immunity, *Macrobrachium nipponense*, norbixin

## Abstract

**Background:**

Natural carotenoids have gained considerable attention in aquaculture due to their potential to enhance growth performance, physiological status, and product quality in crustaceans. However, limited information is available regarding the dietary effects of norbixin on *Macrobrachium nipponense*.

**Objectives:**

This study evaluated the effects of dietary norbixin supplementation on growth performance, haemato‐biochemical indices, immune responses, antioxidant status, digestive enzyme activity, intestinal microbiota, carotenoid accumulation, and body composition in *M. nipponense*.

**Methods:**

A 56‐day feeding trial was conducted using juvenile prawns (initial weight: 1.48 ± 0.07 g) fed diets supplemented with 0.00, 0.05, 0.10, 0.15, and 0.20 g/kg norbixin. Growth indices, biochemical and immunological parameters, antioxidant capacity, digestive enzyme activities, intestinal bacterial populations, tissue carotenoid deposition, and proximate body composition were subsequently assessed.

**Results:**

Dietary norbixin significantly improved growth performance, feed conversion ratio, hepatosomatic index, and survival rate, with the most pronounced effects observed at 0.15 g/kg (*p* < 0.05). Dissolved oxygen levels also increased dose‐dependently and peaked in the 0.15 g/kg treatment (*p* < 0.05). Serum urea, uric acid, glucose, creatinine, cholesterol, and triglyceride concentrations decreased significantly, whereas high‐ and low‐density lipoprotein levels increased (*p* < 0.05). Immune responses and total antioxidant capacity were enhanced, while glutathione peroxidase, alkaline phosphatase, and acid phosphatase activities remained unaffected (*p* > 0.05). Norbixin supplementation further enhanced digestive enzyme activities, increased lactic acid bacteria abundance, reduced total bacterial counts, and promoted carotenoid accumulation in muscle, shell, and hepatopancreas (*p* < 0.05). Whole‐body crude protein and lipid contents increased, whereas moisture and ash contents decreased (*p* < 0.05).

**Conclusions:**

Dietary supplementation with 0.15 g/kg norbixin effectively enhanced growth, immunocompetence, antioxidant defence, intestinal microbial balance, carotenoid deposition, and nutritional quality in *M. nipponense*, suggesting its potential as a functional feed additive in freshwater prawn aquaculture.

## Graphical abstract

Dietary norbixin (0.15 g/kg) optimally promotes growth, immunocompetence, antioxidant defence, gut microbial balance, carotenoid accumulation, and overall nutritional quality in *Macrobrachium nipponense*.

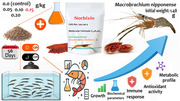

## 1 Introduction

Modern aquaculture has progressively adopted innovative nutritional approaches to maximise productivity and sustainability (Mahfuzur et al. 2018). Within these efforts, customised feeding regimens have become central to promoting the physiological well‐being and adaptability of cultured species (Fawzy et al., 2022a). Nutrient availability directly influences growth trajectories and disease resistance (Pereira da Costa and Campos Miranda‐Filho 2020) while also shaping the tolerance of aquatic organisms to environmental variability (Tan et al. 2024). Although macronutrients are indispensable, the incorporation of functional compounds such as carotenoids has drawn increasing attention for their multifaceted contributions to aquafeed innovation (Sanlier et al. 2024). These fat‐soluble pigments accumulate in body tissues and exert dual functions, both intensifying colouration and acting as bioactive modulators of immunity and development (Yusoff et al. 2020; Fawzy et al., 2022b).

Carotenoids fulfil multiple biological roles, including acting as precursors of vitamins, serving as antioxidants, protecting against UV damage, and improving reproductive efficiency (Yusoff et al. 2020; Fawzy et al., 2022b; Elbahnaswy and Elshopakey 2024). Their antioxidant properties are especially valued for enhancing stress resilience and supporting growth in aquatic organisms (Saidi et al. 2015; Fawzy et al., 2022a; Tan et al. 2024). For these reasons, dietary carotenoids are recognised as critical components of feed formulations that not only improve animal health but also raise product quality and farming efficiency, thereby reinforcing the financial sustainability of aquaculture enterprises (Elbahnaswy and Elshopakey 2024).

In recent years, extensive efforts have been made globally to identify and utilise natural pigments. Compared to many synthetic colourants, natural pigments are generally regarded as safer alternatives; however, their physiological effects remain dose‐dependent, and excessive or inappropriate inclusion levels may still induce adverse outcomes depending on species and exposure conditions. (Mahfuzur et al. 2018; Yusoff et al. 2020; Puri et al. 2025). Annatto, a carotenoid‐based natural colourant, is considered one of the primary global sources of natural food colouring, accounting for approximately 70% of all natural colourants used worldwide. Due to its low cost and ease of application, annatto is widely used in the food industry in products such as beverages, cereals, dairy items, and margarine (Younes et al. 2019; Puri et al. 2025). Annatto extract is derived from the seeds of the *Bixa orellana* L. tree, which is native to the tropical forests of Latin America and is predominantly cultivated in Central and South America as well as parts of Southeast Asia. The reddish‐orange colour of annatto is attributed to the presence of carotenoids located in the seed pericarp, among which norbixin is a prominent compound (Safari and Atash 2015; Younes et al. 2019; Puri et al. 2025). Norbixin, with the chemical formula C_24_H_28_O_4_, is the water‐soluble form of the annatto pigment and is widely utilised as a natural and safe food colourant to impart yellow, orange, and red hues in various food products (Younes et al. 2019; Dananjaya et al. 2020; Dorce et al. 2025).

Because crustaceans cannot synthesise carotenoids endogenously, external supplementation through diet is essential to maintain proper physiological and immune functions (Wade et al., 2017b; Wang et al., 2021; Fawzy et al., 2022b). While astaxanthin continues to be the reference carotenoid in aquaculture, emerging evidence suggests that norbixin can deliver comparable outcomes in growth promotion, immune enhancement, and colouration (Safari and Atash 2015; Dananjaya et al. 2017; Dananjaya et al. 2020; Dorce et al. 2025).

The oriental river prawn (*Macrobrachium nipponense*), belonging to the Palaemonidae family, represents an important freshwater aquaculture species (Fu et al. 2004; Yan et al. 2024). Its popularity stems from valuable production traits, including market demand, adaptability to diverse freshwater environments, omnivorous feeding behaviour, heat tolerance, and lower disease susceptibility compared to other shrimp species (Fu et al. 2004; Kutty 2005; Sun et al. 2016; Tew et al. 2021; Wang et al. 2022; Hooper et al. 2023; Yan et al. 2024). The species is also compatible with formulated diets and exhibits high feed conversion efficiency, highlighting its scalability in aquaculture operations (Zhao et al. 2016; Ding et al. 2017; Ettefaghdoost et al. 2018; Ettefaghdoost and Haghighi 2021; Tian et al. 2023).

Given the established roles of carotenoids in prawn physiology, species‐targeted dietary formulations are required. Previous studies in decapods have reported the benefits of carotenoid supplementation: *β*‐carotene improved development in *Eriocheir sinensis* (Jiang et al. 2024), while astaxanthin and canthaxanthin enhanced immune capacity and growth in *Procambarus clarkii* and *Litopenaeus vannamei*, respectively (Cheng and Wu 2019; Fawzy et al., 2022a).

Building on this evidence, the present study hypothesises that dietary norbixin supplementation can generate synergistic improvements in growth, metabolism, immunity, and tissue characteristics of *M. nipponense*. Despite the well‐recognised effects of carotenoids such as astaxanthin and *β*‐carotene in crustaceans, investigations on norbixin remain scarce, particularly in relation to its broad physiological impacts in this species. Due to the limited availability of systematic studies evaluating norbixin as a functional feed additive in aquaculture species, particularly crustaceans, the present study adopts the broader carotenoid literature as a comparative biochemical and physiological framework. This approach is supported by the structural and redox‐related similarities between norbixin and classical carotenoids, despite differences in formal chemical classification. The objective of this research is to comprehensively evaluate the dose‐dependent effects of norbixin on growth indices, haemato‐biochemical profiles, antioxidant defence, digestive performance, gut microbiota, and carotenoid deposition in juvenile prawns. By integrating diverse parameters within a single framework, this work delivers novel insights connecting norbixin supplementation to aquaculture performance and prawn health. Ultimately, these findings aim to support precision nutrition practices that simultaneously advance productivity, sustainability, and profitability. Given its natural origin, multifunctionality, and cost advantages, norbixin could serve as a promising alternative to synthetic carotenoids, aligning aquaculture production with both economic and ecological goals.

## 2 Materials and Methods

### 2.1 Collection of Prawns and Acclimation Strategy

The present study was carried out over a 56‐day duration at the Sadaf‐Aquatic Research Facility (Rasht, Gilan Province, Iran). Juvenile *M. nipponense* were sourced from the Hend Khaleh River, a key freshwater ecosystem within the southern Caspian Sea basin (situated 16 m below sea level; coordinates: 37°22′55″N, 49°26′39″E). Following collection, prawns weighing between 1.0 and 1.5 g, with lengths ranging from 5.0 to 5.5 cm, were acclimated for 14 days in a 1000‐L fibreglass tank (Sinta, FRP model, Hebei, China). During acclimation, specimens were fed a species‐specific diet comprising 44–45% protein, 4–5% lipids, 13–14% ash, 9–10% moisture, and an energy density of 18 kJ/g (Ettefaghdoost et al. 2018). Feed particle size was standardised at 1 mm to correspond with the oral morphology of the prawns.

### 2.2 Experimental Setup and Diet Formulation

After the acclimation phase, individual body weights and lengths of prawns were measured using a digital balance (A&D, model EK‐2000i, Tokyo, Japan) and a precision digital caliper (Mitutoyo, model 500‐196‐30, Kawasaki, Japan). The animals were then randomly allocated into five dietary treatments (average weight: 1.48 ± 0.07 g; average length: 5.27 ± 0.09 cm), each consisting of three replicates with 25 prawns (13 males ♂ and 12 females ♀) per replicate. A total of 15 glass aquaria (90 × 35 × 40 cm; 8 mm thickness) were used, each stocked with 100 L of dechlorinated water. Water quality was maintained through partial daily renewal (approximately one‐third of the total volume) and complete replacement at each sampling point. Continuous aeration was supplied using a central air blower (Resun, model ACO‐009D, Shenzhen, China). All tanks were maintained under identical aeration, water exchange, and stocking conditions. Dissolved oxygen levels were monitored regularly and did not differ significantly among treatments throughout the trial. Lighting conditions followed a 12 h light:12 h dark cycle, maintained with LED lamps (Philips, model Essential LED‐tube, Amsterdam, Netherlands).

Dietary formulations were generated using GAMS optimisation software (version 35.1, Washington, USA). Feed ingredients were finely milled (IKA, model MF 10.2, Staufen, Germany), sieved through a 100 µm stainless steel mesh (Endecotts, model EFL 2000, London, UK), weighed, and thoroughly blended until a dough‐like texture was achieved. The homogenised mixture was extruded into 1 mm strands using a laboratory‐scale pelletiser (Clextral, model BC 21, Firminy, France).

Norbixin powder (TonKing Biotech, Norbixin CWS/S‐TG 10%, CAS No. 542‐40‐5, Cat. No. TK‐2406, E160b, Xi'an, China), derived from annatto (*Bixa orellana* L.), was solubilised in distilled water and applied by spray‐coating onto the pellets. Coated diets were dried using a forced‐air oven (Memmert, model UF55, Schwabach, Germany) and stored at −18°C in a laboratory freezer (Thermo Fisher Scientific, model TSX Series, Waltham, USA). Daily feed portions were kept at 4°C in light‐protected polyethylene containers (Kartell, model 231, Noviglio, Italy) to prevent oxidative degradation. Five dietary treatments were prepared with graded norbixin inclusion levels of 0.0 (control), 0.05, 0.10, 0.15, and 0.20 g/kg. Prawns were hand‐fed four times daily (08:00, 12:00, 16:00, 20:00) at a feeding rate of 3% of body weight (Ettefaghdoost et al. 2018). Feed portions were weighed using a high‐precision balance (Sartorius, model Cubis II, Göttingen, Germany) with 0.01 g accuracy (Ettefaghdoost and Haghighi 2021). Although a specific leaching rate assay was not performed, feed preparation and management practices were designed to minimise nutrient loss. Future studies should quantify norbixin leaching dynamics under controlled immersion conditions to further refine dietary application strategies. The ingredient composition of the experimental diets is summarised in Table 1, prepared according to AOAC (2016) standards.

**TABLE 1:** Formulation and nutrient profile of experimental diets (% dry matter basis) used in this study.

	Norbixin (g/kg)				
	0.0	0.05	0.10	0.15	0.20
Ingredients (%)					
Fish powder^a^	30.00	30.00	30.00	30.00	30.00
Soy flour	30.00	30.00	30.00	30.00	30.00
Wheat flour	7.00	7.00	7.00	7.00	7.00
Corn flour	7.00	7.00	7.00	7.00	7.00
Casein^b^	16.00	16.00	16.00	16.00	16.00
Vitamix^c^	2.00	2.00	2.00	2.00	2.00
Minmix^d^	2.00	2.00	2.00	2.00	2.00
Cholesterol^e^	0.20	0.20	0.20	0.20	0.20
Ascorbate^f^	0.10	0.10	0.10	0.10	0.10
DCP^g^	0.50	0.50	0.50	0.50	0.50
Filler (CMC) premix^h^	5.20	5.195	5.19	5.185	5.18
Norbixin^i^	0.00	0.005	0.01	0.015	0.020
Nutritional composition					
Moisture (%)	9.70	9.83	9.64	9.78	9.61
Crude protein (%)	45.03	45.10	44.86	45.07	44.92
Crude lipid (%)	5.06	5.11	5.07	5.13	5.04
Fibre (%)	2.97	3.06	3.02	3.02	3.00
Ash (%)	12.97	13.08	12.91	13.02	12.95
Nitrogen free extract (%)	24.27	23.82	24.50	23.98	24.48
Gross energy (kJ/g)^j^	19.83	19.90	19.88	19.75	19.97
Total carotenoids (mg/kg)	4.82	52.90	107.61	149.23	202.11

^a^ Arad‐Powder, Tehran, Iran.

^b^ Quelab Inc., Montréal, Canada (CAS No. 9000‐71‐9, EC No. 232‐555‐1).

^c^ Aras Pharmaceutical Co., Tehran, Iran—Aquavit‐1 vitamin premix (1000 g) comprising vitamin A (retinol) 1,200,000 IU, vitamin B1 (thiamine) 2500 mg, vitamin B2 (riboflavin) 4000 mg, vitamin B6 (pyridoxine) 2500 mg, vitamin B7 (biotin) 150 mg, vitamin B9 (folate) 1000 mg, vitamin B12 (cobalamin) 8 mg, vitamin C (ascorbic acid) 30,000 mg, calcium pantothenate 10,000 mg, vitamin D3 (cholecalciferol) 400,000 IU, vitamin K3 (menadione) 800 mg, niacin (nicotinic acid) 35,000 mg.

^d^ Sci Laboratories Inc., Qazvin, Iran—mineral premix (1000 g) containing cobalt (Co) 100 mg, copper (Cu) 600 mg, choline chloride 6000 mg, Iodine (I) 600 mg, iron (Fe) 6000 mg, manganese (Mn) 5000 mg, selenium (Se) 20 mg, zinc (Zn) 10,000 mg.

^e^ Merck Group, Darmstadt, Germany (CAS No. 57‐88‐5, EC No. 200‐353‐2).

^f^ Aras Pharmaceutical Co., Tehran, Iran—Aquavit‐C vitamin C premix (500 g) with Stay‐C 35%.

^g^ Aras Pharmaceutical Co., Tehran, Iran (CAS No. 7757‐93‐9, EC No. 231‐826‐1).

^h^ Kimia‐Lab Inc., Tehran, Iran.

^i^ TonKing Biotech, Xi'an, China—CWS/S‐TG 10% (CAS No. 542‐40‐5, Cat. No. TK‐2406, E160b).

^j^ Energy values per gram: protein 16.7 kJ, lipid 37.6 kJ, carbohydrate 16.7 kJ.

### 2.3 Water Quality Monitoring and Sampling Procedures

Key water parameters including temperature, pH, and dissolved oxygen (DO) were measured daily using a portable multi‐parameter meter (Hach, model HQ440D, Loveland, USA). Concentrations of total ammonia nitrogen (TAN), nitrite, and nitrate were determined twice weekly through spectrophotometric methods with a benchtop UV–Vis spectrophotometer (Jenway, model 7315, Staffordshire, UK), following standardised analytical protocols.

At the completion of the 56‐day feeding trial, prawns were fasted for 48 h prior to sampling (Gu et al. 2017). From each replicate aquarium, 10 individuals were randomly selected for analysis. Final body weights and lengths were recorded, and growth performance indices were calculated as follows (Huang et al. 2019; Tian et al. 2023):

$$\text{Weight gain}\left(\text{WG},g\right)=W_{\mathrm{final}(g)}-W_{\mathrm{initial}(g)},$$

$$\text{Weight gain rate}\left(\text{WGR},\%\right)=100\times \left(WG_{\left(g\right)}÷W_{\mathrm{initial}(g)}\right),$$

$$\begin{array}{c} \text{Specific growth rate}(\text{SGR},\%/\text{day})\\ =100\times \left[\left(\ln W_{\mathrm{final}(g)}\right)\text{--}\ln W_{\mathrm{initial}(g)}\right)÷\text{experimental}{\text{period}}_{\left(\mathrm{days}\right)}], \end{array}$$

$$\text{Feed conversion ratio}\left(\text{FCR}\right)=\text{feed}{\text{intake}}_{\left(g\right)}÷WG_{\left(g\right)},$$

$$\begin{array}{c} \text{Hepatosomatic index}(\text{HSI},\%)\\ =100\times (W_{\mathrm{hepatopancreas}(g(}÷W_{\mathrm{total}\mathrm{body}(g)}), \end{array}$$

$$\text{Survival rate}\left(\text{SR},\%\right)=100\times \left(N_{\mathrm{final}}÷N_{\mathrm{initial}}\right)$$

Haemolymph samples were collected using 1 mL sterile syringes (Terumo, model SS‐01T, Tokyo, Japan) from the ventral sinus (Niu et al. 2009). Samples were centrifuged at 12,000 rpm for 15 min at 4°C in a refrigerated centrifuge (Eppendorf, model 5418R, Hamburg, Germany) to separate serum (Zhao et al. 2016). Hepatopancreas and muscle tissues were carefully excised using sterile dissection kits (VWR, model 82027–460, Radnor, USA), rinsed in isotonic saline, and immediately frozen in liquid nitrogen (Chien and Shiau 2005). All biological samples were stored at −80°C pending biochemical assays (Tian et al. 2023).

### 2.4 Biochemical Assays

The pooled specimens were gradually thawed at 4°C prior to biochemical assessment. Analyses were performed using an automated clinical chemistry analyser (Mindray, model BS‐240, Shenzhen, China) in conjunction with commercial colorimetric reagent kits (Abcam, Cambridge, UK). All assays were carried out at room temperature (22 ± 1°C), and duplicate measurements were obtained to ensure reproducibility. Quality control procedures included the use of reagent blanks, calibration curves, intra‐assay variation checks (<5%), and the incorporation of standard reference controls as described by Xu et al. (2019) and Yang et al. (2023).

### 2.5 Assessment of Haematological and Immune Biomarkers

Serum albumin (ALB) concentration was determined by the Bromocresol Green (BCG) colorimetric method, while total protein (TP) was quantified using the Biuret reaction, both with commercial diagnostic kits (BioSystems, Cat. Nos. ALB‐11515 and TP‐11528, Barcelona, Spain) at a wavelength of 546 nm (Kuo et al. 2019). Cortisol (CORT) levels were measured using a competitive ELISA kit (Enzo Life Sciences, Cat. No. ADI‐900‐071, Farmingdale, USA).

Lysozyme (LYZ) activity was assessed by the turbidimetric assay based on the lysis of *Micrococcus luteus* (ATCC 4698, Kwik‐Stik, Hardy Diagnostics, Cat. No. 0804K, California, USA), with absorbance recorded at 450 nm using an ELISA plate reader (BMG Labtech, model CLARIOstar Plus, Ortenberg, Germany). Phenoloxidase (PO) activity was quantified spectrophotometrically at 490 nm by monitoring DOPA‐chrome formation (Liu et al. 2019).

For total haemocyte counts (THC), 0.1 mL of haemolymph was mixed with an anticoagulant solution and 10% neutral‐buffered formalin, followed by a 30 min incubation (Xu et al. 2019). Cells were enumerated with a haemocytometer (Marienfeld Superior, model Neubauer‐Improved, Lauda‐Königshofen, Germany) under a compound light microscope (Olympus, model CX23, Tokyo, Japan) at 40× magnification. Differential haemocyte counts (DHC) were performed by preparing haemolymph smears, fixing with absolute methanol (Fisher Chemical, Cat. No. A452, Loughborough, UK), and staining with 10% Giemsa solution (Thermo Fisher Scientific, Cat. No. R04010, Waltham, USA). Slides were air‐dried and examined microscopically to classify haemocyte subtypes based on granule morphology (Tian et al. 2023).

Enzymatic activities including acid phosphatase (ACP), lactate dehydrogenase (LDH), aspartate aminotransferase (AST), alanine aminotransferase (ALT), and alkaline phosphatase (AKP) were measured using diagnostic kits (Randox Laboratories, Crumlin, UK). Readings were obtained in the range of 340–405 nm on the automated biochemistry analyser (Xu et al. 2019; Yang et al. 2023).

### 2.6 Determination of Antioxidant Defence Markers

For antioxidant assessment, 10 prawns from each replicate were randomly selected, and hepatopancreatic tissues were dissected on ice. Samples were rinsed in chilled double‐distilled water (Zolal‐Teb Shimi, Tehran, Iran), weighed, and homogenised at a 1:9 (w/v) ratio in Tris‐HCl buffer (TCI Chemicals, Cat. No. T0932, Tokyo, Japan) as described by Zhang et al. (2013). Homogenisation was performed with a laboratory homogeniser (Heidolph, model Silent‐Crusher M, Schwabach, Germany), followed by centrifugation at 12,000 rpm for 15 min at 4°C in a refrigerated centrifuge (Thermo Scientific, model Sorvall ST 8R, Waltham, USA). The resulting supernatants were aliquoted into sterile cryovials and stored at −80°C until biochemical analysis (Han et al. 2018).

Antioxidant biomarkers were quantified using commercial colorimetric kits (Cayman Chemical, Ann Arbor, USA), including malondialdehyde (MDA; Cat. No. 10009055), glutathione peroxidase (GPx; Cat. No. 703102), superoxide dismutase (SOD; Cat. No. 706002), catalase (CAT; Cat. No. 707002), and total antioxidant capacity (T‐AOC; Cat. No. 709001). Absorbance values were recorded with a microplate spectrophotometer (BMG Labtech, model CLARIOstar Plus, Ortenberg, Germany) according to the manufacturer's protocols (Tian et al. 2023).

### 2.7 Evaluation of Digestive Enzyme Activities

To investigate digestive enzyme function, prawns were starved for 48 h to eliminate intestinal contents. Ten specimens from each replicate were then randomly sampled, and intestinal tissues were dissected on ice to preserve enzymatic activity. Samples were rinsed with chilled double‐distilled water, blotted dry, weighed on an analytical balance (Mettler Toledo, model ME204E, Greifensee, Switzerland), and homogenised in a 1:9 (w/v) ratio with Tris‐HCl buffer following the method of Wang et al. (2018). Homogenisation was performed using a laboratory homogeniser, and the homogenates were centrifuged at 10,000 rpm for 10 min at 4°C in a refrigerated centrifuge. The supernatant fractions were collected in sterile tubes and stored at −80°C until enzyme assays were conducted (Weilong et al. 2019).

The activities of protease, amylase, cellulase, and lipase were determined spectrophotometrically using commercial diagnostic kits (Megazyme, Bray, Ireland): Protease Activity Assay Kit (Cat. No. K‐PR001), Amylase Activity Assay Kit (Cat. No. K‐AMYL), Cellulase Activity Assay Kit (Cat. No. K‐CELL), and Lipase Activity Assay Kit (Cat. No. K‐LIPGL). Absorbance readings were taken at 366 nm (protease), 405 nm (amylase), 540 nm (cellulase), and 550 nm (lipase), following the manufacturer's protocols and established methodologies (Ghosh 2023).

### 2.8 Determination of Intestinal Microbiota

To evaluate the intestinal microbial community, 10 prawns from each experimental group (randomly 4–3–3 distribution per replicate) were first anaesthetised using an ice‐slurry technique. The ventral abdominal area was sterilised with 70% ethanol (Foonoon‐Teb, Tehran, Iran). Under aseptic conditions within a Class II biosafety cabinet, intestines were carefully excised, slit longitudinally, and washed three times with sterile physiological saline (Thermo Fisher Scientific, Cat. No. 12345, Waltham, USA).

The intestinal contents were then serially diluted from 10^−^^1^ to 10^−^^10^ using sterile saline. Aliquots of 0.1 mL from each dilution were plated onto nutrient‐rich tryptic soy agar (TSA) (HiMedia, Cat. No. M1112, Mumbai, India) to enumerate total heterotrophic bacteria, and onto De Man–Rogosa–Sharpe (MRS) agar (HiMedia, Cat. No. M641, Mumbai, India) for quantification of lactic acid bacteria. Plates were incubated at 32 ± 2°C for 48–72 h, after which colonies were counted using a digital colony counter (Cole‐Parmer, model SC6PLUS, Vernon Hills, USA).

Microbial counts were expressed as logarithmic colony‐forming units per gram of intestinal material (log CFU/g). All analyses were performed in triplicate to ensure precision and reproducibility, following modified protocols from earlier studies (Miao et al. 2017; Lu et al. 2023; Luo et al. 2023).

### 2.9 Quantitative Analysis of Total Carotenoids in Prawn Tissue

The total carotenoid content in different prawn tissues—including shell, hepatopancreas, and muscle—was determined using a spectrophotometric method. For each sample, 1 g of tissue was homogenised in 10 mL of acetone (Fisher Chemical, Cat. No. A998‐500, ≥98% purity, Loughborough, UK) together with 2 g of anhydrous sodium sulphate (Alfa Aesar, Cat. No. L12345, Haverhill, USA). The mixture was homogenised for 15 min and subsequently filtered through qualitative filter paper (Advantec, Grade 5, Tokyo, Japan). The extraction procedure was repeated three times, and the combined extracts were centrifuged at 3500 rpm for 15 min using a benchtop centrifuge (Eppendorf, model 5424R, Hamburg, Germany). The absorbance of the clear supernatant was measured at 450 nm with a UV–Vis spectrophotometer (Agilent, model Cary 60, Santa Clara, USA). Carotenoid concentrations were calculated using an extinction coefficient of 2500 and expressed as micrograms per gram of tissue (µg/g) (Hu et al. 2019; Fawzy et al., 2022b).

### 2.10 Comprehensive Analysis of Prawn Carcass Composition

Proximate analysis of prawn carcasses was performed following AOAC (2016) guidelines. Moisture content was assessed by placing 5 g of homogenised tissue into pre‐dried Petri dishes (Sarstedt, Product No. 83.3900, Nümbrecht, Germany), followed by oven drying at 103 ± 2°C until a constant weight was reached using a convection oven (Yamato, model DHG‐9140A, Tokyo, Japan). Dried samples were cooled in a desiccator (Labconco, model SD‐DES01, Kansas City, USA) prior to final weighing. Crude protein was determined using the Kjeldahl method with a nitrogen analyser (Kjeltec, model k9840, Hillerød, Denmark). Lipid content was extracted via the Soxhlet method (Buchi, model sox406, Flawil, Switzerland) using chloroform (Mojallali, Tehran, Iran) as solvent. Ash content was measured by incinerating samples at 550°C for 12 h in a muffle furnace (Nabertherm, model SX2‐2.5‐10NP, Lilienthal, Germany). All proximate analyses were carried out in triplicate and reported as percentages of wet body weight. Quality control included reagent blanks, certified reference materials, and within‐run repeatability thresholds (<3% variation) (Gu et al. 2017; Fan et al. 2018; Liou et al. 2023; Lu et al. 2023).

### 2.11 Statistical Analysis

Statistical analyses were performed using IBM SPSS Statistics (version 27.0.1; Armonk, NY, USA). Normality of data was assessed with the Kolmogorov–Smirnov test, and homogeneity of variances was examined using Levene's test. Differences among treatment groups were evaluated via one‐way ANOVA, followed by pairwise comparisons with Duncan's multiple range test. Furthermore, orthogonal polynomial regression was applied to explore potential linear and quadratic trends. Statistical significance was defined at *p* < 0.05. Data are presented as mean ± standard deviation.

## 3 Results

### 3.1 Water Quality Parameters

Dietary norbixin supplementation did not exert significant effects on water temperature, pH, ammonium, nitrite, nitrate, phosphate, or total dissolved solids (*p* > 0.05, Table 2). Conversely, dissolved oxygen levels showed a significant improvement (*p* < 0.05), increasing from 7.05 mg/L in the control group to 7.76 mg/L at 0.15 g/kg norbixin inclusion (*p* < 0.05).

**TABLE 2:** Effects of norbixin‐enriched diets on *Macrobrachium nipponense*: assessment of water quality indicators during a 56‐day feeding trial.

	Norbixin (g/kg)					ANOVA	Linear	Quadratic
Parameters	0.0	0.05	0.10	0.15	0.20	*p*‐value (*F*)	*p*‐value (*R* ^2^)	*p*‐value (*R* ^2^)
Temperature (°C)	25.61 ± 0.54	26.54 ± 0.40	25.37 ± 0.38	25.40 ± 0.59	25.52 ± 0.40	0.863 (0.311)	0.152 (0.595)	0.216 (0.772)
pH	7.15 ± 0.23	7.59 ± 0.18	7.12 ± 0.34	7.24 ± 0.19	7.18 ± 0.22	0.618 (0.747)	0.799 (0.325)	0.593 (0.422)
Dissolved oxygen (mg/L)	7.05 ± 0.13^d^	7.68 ± 0.05^ab^	7.25 ± 0.07^c^	7.76 ± 0.04^a^	7.52 ± 0.06^b^	<0.001 (29.841)	0.037 (0.986)	0.031 (0.967)
Ammonium (mg/L)	0.46 ± 0.04	0.44 ± 0.04	0.46 ± 0.05	0.42 ± 0.03	0.42 ± 0.06	0.529 (0.920)	0.388 (0.265)	0.752 (0.277)
Nitrite (mg/L)	0.13 ± 0.02	0.13 ± 0.04	0.12 ± 0.02	0.13 ± 0.03	0.12 ± 0.02	0.552 (0.844)	0.174 (0.523)	0.203 (0.758)
Nitrate (mg/L)	0.15 ± 0.02	0.16 ± 0.02	0.17 ± 0.02	0.15 ± 0.01	0.16 ± 0.02	0.421 (1.186)	0.586 (0.113)	0.213 (0.852)
Phosphate (mg/L)	0.03 ± 0.01	0.03 ± 0.02	0.02 ± 0.02	0.02 ± 0.02	0.03 ± 0.01	0.459 (0.898)	0.323 (0.756)	0.171 (0.846)
Total dissolved solids (mg/L)	165.45 ± 3.95	168.49 ± 2.75	166.28 ± 3.30	160.69 ± 2.81	162.53 ± 2.88	0.327 (1.562)	0.169 (0.505)	0.476 (0.544)

*Note*: Values represent mean ± standard deviation (*M* ± *SD*, *n* = 3). Different superscripts in the same raw with values ranging from a to e indicate a significant difference (*p* < 0.05).

### 3.2 Growth Performance

**FIGURE 1:**
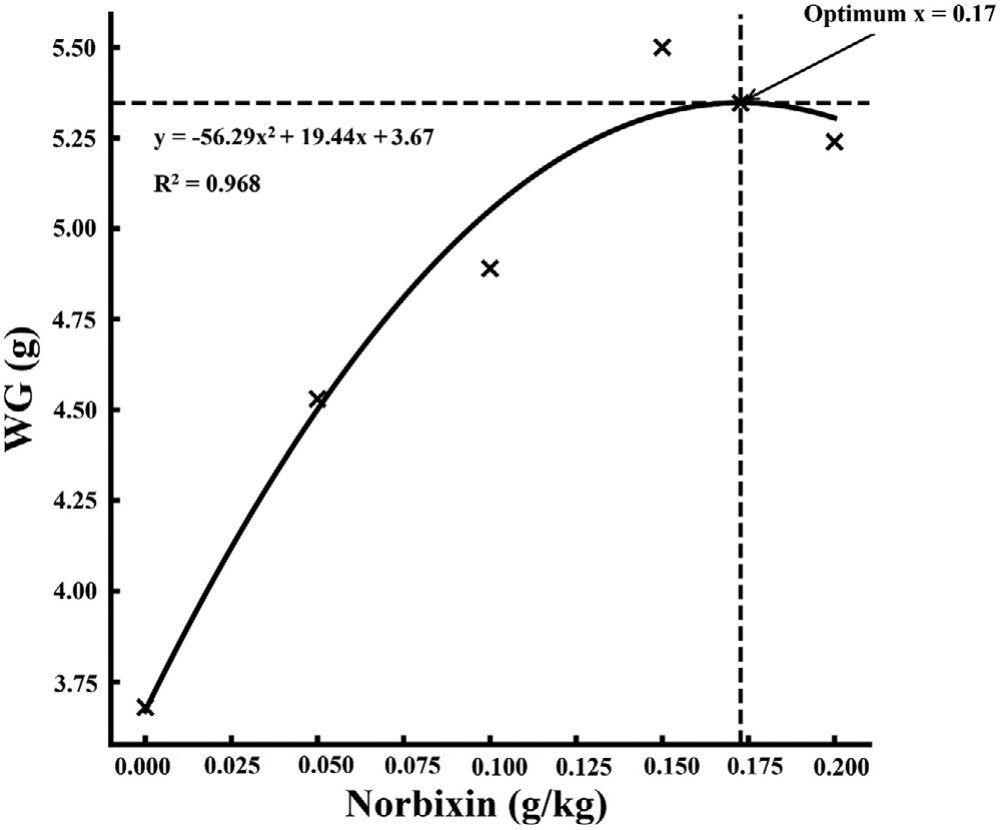
Quadratic regression of weight gain (WG) in *Macrobrachium nipponense* fed norbixin‐supplemented diets over 56 days.

**FIGURE 2:**
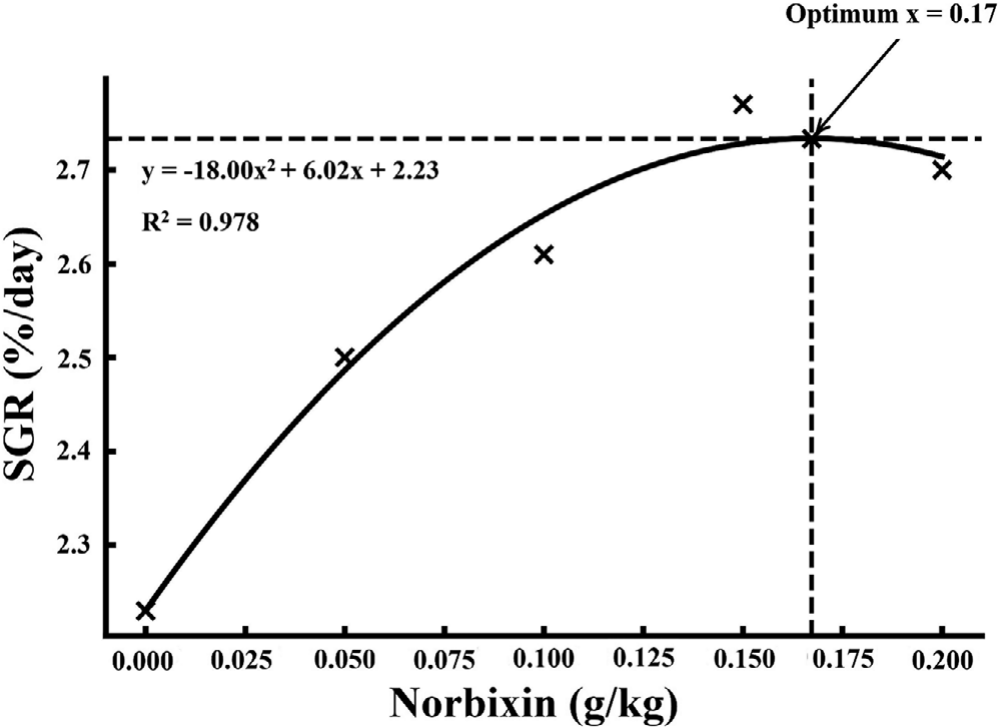
Quadratic regression of specific growth rate (SGR) in *Macrobrachium nipponense* fed norbixin‐supplemented diets over 56 days.

**FIGURE 3:**
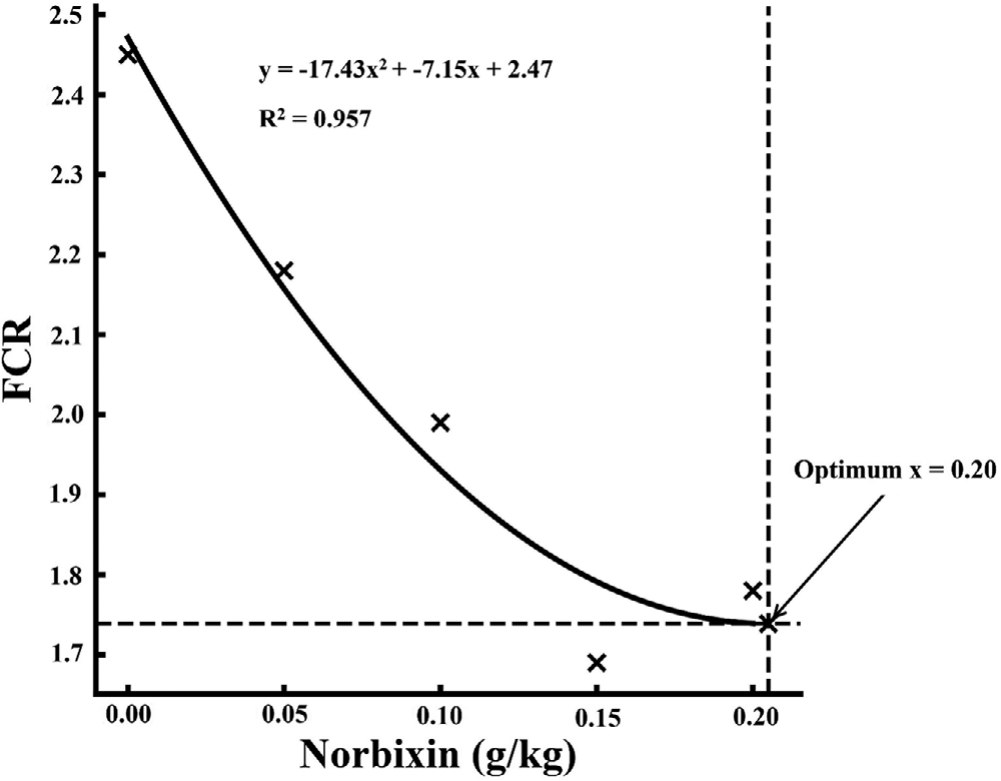
Quadratic regression of feed conversion ratio (FCR) in *Macrobrachium nipponense* fed norbixin‐supplemented diets over 56 days.

All growth performance indices were significantly enhanced by dietary norbixin supplementation (*p* < 0.05, Table 3). FW increased from 5.13 g in the control group to a maximum of 6.98 g at 0.15 g/kg inclusion. Similarly, WG rose from 3.68 g to 5.50 g, while WGR improved from 248.65% to 371.62%. SGR increased from 2.23%/day to 2.77%/day at the highest inclusion levels. In contrast, FCR significantly decreased from 2.45 in the control group to 1.69 at 0.15 g/kg. HSI also increased progressively, peaking at 5.19%, while SR reached 100% at 0.15 g/kg. Regression analyses for WG, SGR, and FCR demonstrated statistically significant quadratic trends (*p* < 0.05, Figures 1, 2, 3).

**TABLE 3:** Effects of norbixin‐enriched diets on *Macrobrachium nipponense*: assessment of growth performance during a 56‐day feeding trial.

	Norbixin (g/kg)					ANOVA	Linear	Quadratic
Parameters	0.0	0.05	0.10	0.15	0.20	*p*‐value (*F*)	*p*‐value (*R* ^2^)	*p*‐value (*R* ^2^)
Final weight (g)	5.13 ± 0.38^e^	6.01 ± 0.37^d^	6.37 ± 0.36^c^	6.98 ± 0.32^a^	6.72 ± 0.29^b^	<0.001 (231.370)	0.045 (0.938)	0.020 (0.959)
Weight gain (g)	3.68 ± 0.30^e^	4.53 ± 0.27^d^	4.89 ± 0.36^c^	5.50 ± 0.41^a^	5.24 ± 0.33^b^	<0.001 (253.152)	0.013 (0.955)	0.033 (0.968)
Weight gain rate (%)	248.65 ± 23.61^e^	306.08 ± 25.70^d^	330.41 ± 28.22^c^	371.62 ± 19.82^a^	354.05 ± 29.18^b^	<0.001 (121.289)	0.021 (0.962)	0.018 (0.951)
Specific growth rate (%/day)	2.23 ± 0.17^d^	2.50 ± 0.15^c^	2.61 ± 0.19^b^	2.77 ± 0.12^a^	2.70 ± 0.11^ab^	<0.001 (194.662)	0.038 (0.811)	0.035 (0.978)
Feed conversion ratio	2.45 ± 0.06^a^	2.18 ± 0.07^b^	1.99 ± 0.05^c^	1.69 ± 0.07^e^	1.78 ± 0.04^d^	<0.001 (411.259)	0.041 (0.824)	0.005 (0.957)
Hepatosomatic index (%)	4.51 ± 0.42^d^	4.96 ± 0.58^c^	5.07 ± 0.49^b^	5.19 ± 0.39^a^	5.03 ± 0.37^b^	<0.001 (210.874)	0.043 (0.892)	0.025 (0.935)
Survival rate (%)	92.73 ± 3.85^d^	95.48 ± 2.74^c^	98.33 ± 1.40^b^	100.00 ± 0.00^a^	98.82 ± 1.16^b^	<0.001 (463.819)	0.030 (0.968)	0.028 (0.947)

*Note*: Values represent mean ± standard deviation (*M* ± *SD*, *n* = 3). Different superscripts in the same raw with values ranging from a to e indicate a significant difference (*p* < 0.05).

### 3.3 Haemato‐Biochemical Indices

Dietary norbixin supplementation significantly influenced several haemato‐biochemical parameters in *M. nipponense* (*p* < 0.05, Table 4). Concentrations of urea, uric acid, glucose, and creatinine declined progressively with increasing norbixin levels, reaching minimum values of 16.46 mg/dL, 1.63 mg/dL, 51.84 mg/dL, and 0.29 mg/dL, respectively, at 0.15 g/kg. Conversely, HDL and LDL concentrations increased significantly, peaking at 18.43 mg/dL and 9.95 mg/dL, respectively, in prawns fed 0.15 g/kg norbixin. In contrast, cholesterol and triglyceride levels decreased markedly, with the lowest values observed at 0.15 g/kg (44.68 mg/dL and 68.62 mg/dL, respectively). No significant variations were detected in calcium and phosphorus concentrations (*p* > 0.05).

**TABLE 4:** Effects of norbixin‐enriched diets on *Macrobrachium nipponense*: assessment of haemato‐biochemical parameters during a 56‐day feeding trial.

	Norbixin (g/kg)					ANOVA	Linear	Quadratic
Parameters	0.0	0.05	0.10	0.15	0.20	*p*‐value (*F*)	*p*‐value (*R* ^2^)	*p*‐value (*R* ^2^)
Urea (mg/dL)	18.51 ± 0.62^a^	17.49 ± 0.41^b^	17.17 ± 0.28^c^	16.46 ± 0.30^e^	16.94 ± 0.37^d^	<0.001 (33.499)	0.037 (0.894)	0.303 (0.889)
Uric acid (mg/dL)	1.92 ± 0.25^a^	1.77 ± 0.18^b^	1.75 ± 0.22^b^	1.63 ± 0.09^d^	1.68 ± 0.12^c^	<0.001 (18.245)	0.024 (0.817)	0.022 (0.828)
Glucose (mg/dL)	54.68 ± 0.59^b^	55.87 ± 0.42^a^	54.47 ± 0.53^b^	51.84 ± 0.29^d^	53.84 ± 0.24^c^	<0.001 (105.997)	0.034 (0.918)	0.021 (0.953)
Creatinine (mg/dL)	0.35 ± 0.04^a^	0.34 ± 0.02^ab^	0.33 ± 0.02^b^	0.29 ± 0.01^c^	0.30 ± 0.01^bc^	0.008 (8.378)	0.003 (0.955)	0.002 (0.959)
Calcium (mg/dL)	80.63 ± 1.94	83.19 ± 1.95	79.82 ± 1.89	80.30 ± 1.35	80.24 ± 1.51	0.803 (0.641)	0.145 (0.584)	0.318 (0.675)
Phosphorus (mg/dL)	19.13 ± 0.69	19.76 ± 0.59	19.20 ± 0.82	19.02 ± 0.67	18.95 ± 0.42	0.965 (0.196)	0.247 (0.391)	0.341 (0.623)
Cholesterol (mg/dL)	49.06 ± 0.59^b^	49.86 ± 0.38^a^	45.35 ± 0.32^c^	44.68 ± 0.28^d^	45.39 ± 0.27^c^	<0.001 (130.625)	0.003 (0.928)	0.002 (0.931)
Triglycerides (mg/dL)	78.77 ± 0.73^a^	76.21 ± 0.52^b^	75.03 ± 0.43^c^	68.62 ± 0.28^e^	74.43 ± 0.32^d^	<0.001 (128.62)	0.030 (0.852)	0.029 (0.865)
High‐density lipoprotein (mg/dL)	15.45 ± 0.35^d^	16.86 ± 0.38^c^	16.82 ± 0.25^c^	18.43 ± 0.29^a^	17.46 ± 0.27^b^	<0.001 (24.073)	0.027 (0.892)	0.019 (0.915)
Low‐density lipoprotein (mg/dL)	8.07 ± 0.17^e^	8.73 ± 0.25^d^	9.11 ± 0.23^c^	9.95 ± 0.19^a^	9.83 ± 0.21^b^	<0.001 (54.103)	0.016 (0.920)	0.005 (0.918)

*Note*: Values represent mean ± standard deviation (*M* ± *SD*, *n* = 3). Different superscripts in the same raw with values ranging from a to e indicate a significant difference (*p* < 0.05).

### 3.4 Haemato‐Immune Responses

Dietary norbixin supplementation markedly modulated haemato‐immune responses in *M. nipponense* (*p* < 0.05, Table 5). ALB and TP levels increased significantly, with maximum values of 2.17 g/dL and 10.01 g/dL, respectively, observed at 0.15 g/kg inclusion. Conversely, CORT concentrations declined progressively, reaching the lowest level (14.77 ng/mL) at 0.15 g/kg, indicating reduced stress response. Immune enzyme activities also responded positively to norbixin; LYZ activity peaked at 22.41 U/min/mL, and PO reached 1.12 U/min/mg protein in the 0.15 g/kg group (*p* < 0.05).

**TABLE 5:** Effects of norbixin‐enriched diets on *Macrobrachium nipponense*: assessment of haemato‐immune indices during a 56‐day feeding trial.

	Norbixin (g/kg)					ANOVA	Linear	Quadratic
Parameters	0.0	0.05	0.10	0.15	0.20	*p*‐value (*F*)	*p*‐value (*R* ^2^)	*p*‐value (*R* ^2^)
Albumin (g/dL)	1.72 ± 0.03^c^	1.95 ± 0.05^b^	1.97 ± 0.02^b^	2.17 ± 0.05^a^	1.97 ± 0.04^b^	<0.001 (62.934)	0.041 (0.774)	0.030 (0.772)
Total protein (g/dL)	7.36 ± 0.17^d^	8.39 ± 0.12^c^	8.36 ± 0.32^c^	10.01 ± 0.15^a^	9.33 ± 0.18^b^	<0.001 (223.878)	0.034 (0.856)	0.027 (0.968)
Cortisol (ng/mL)	20.15 ± 0.72^a^	17.11 ± 0.57^b^	16.26 ± 0.42^c^	14.77 ± 0.33^e^	15.68 ± 0.41^d^	<0.001 (103.174)	0.044 (0.889)	0.031 (0.910)
Lysozyme (U/min/mL)	17.11 ± 0.34^e^	18.29 ± 0.39^d^	20.73 ± 0.28^b^	22.41 ± 0.33^a^	19.83 ± 0.40^c^	<0.001 (295.176)	0.015 (0.897)	0.007 (0.947)
Phenoloxidase (U/min/mg protein)	0.80 ± 0.04^d^	0.86 ± 0.07^c^	0.96 ± 0.03^b^	1.12 ± 0.04^a^	0.91 ± 0.02^bc^	<0.001 (19.259)	0.013 (0.929)	0.009 (0.912)

*Note*: Values represent mean ± standard deviation (*M* ± *SD*, *n* = 3). Different superscripts in the same raw with values ranging from a to e indicate a significant difference (*p* < 0.05).

### 3.5 Cell‐Mediated Immune Responses

Dietary norbixin supplementation significantly enhanced cell‐mediated immune parameters (*p* < 0.05, Table 6). THC, GC, SGC, and HC values all increased with dietary treatments, reaching maximum levels of 157.13 ×10^5^ cells/mL, 29.16 ×10^5^ cells/mL, 63.18 ×10^5^ cells/mL, and 65.97 ×10^5^ cells/mL, respectively, at 0.15 g/kg (*p* < 0.05).

**TABLE 6:** Effects of norbixin‐enriched diets on *Macrobrachium nipponense*: assessment of cell‐mediated immune responses during a 56‐day feeding trial.

	Norbixin (g/kg)					ANOVA	Linear	Quadratic
Parameters	0.0	0.05	0.10	0.15	0.20	*p*‐value (*F*)	*p*‐value (*R* ^2^)	*p*‐value (*R* ^2^)
Total haemocyte count (×10^5^ cells/mL)	106.78 ± 2.08^e^	122.03 ± 1.89^d^	141.68 ± 1.75^c^	157.13 ± 1.82^a^	151.96 ± 1.21^b^	<0.001 (283.691)	0.039 (0.837)	0.011 (0.974)
Granular cells (×10^5^ cells/mL)	13.94 ± 1.58^e^	20.61 ± 2.14^d^	23.25 ± 1.21^c^	29.16 ± 1.32^a^	24.64 ± 1.25^b^	<0.001 (48.423)	0.029 (0.793)	0.025 (0.967)
Semi‐granular cells (×10^5^ cells/mL)	42.56 ± 3.09^e^	47.13 ± 2.37^d^	56.11 ± 1.09^c^	63.18 ± 1.24^a^	62.78 ± 1.16^b^	<0.001 (82.507)	0.038 (0.875)	0.018 (0.936)
Hyaline cells (×10^5^ cells/mL)	49.91 ± 3.21^e^	56.12 ± 2.99^d^	56.76 ± 1.42^c^	65.97 ± 1.38^a^	65.05 ± 1.44^b^	<0.001 (106.523)	0.034 (0.812)	0.013 (0.968)

*Note*: Values represent mean ± standard deviation (*M* ± *SD*, *n* = 3). Different superscripts in the same raw with values ranging from a to e indicate a significant difference (*p* < 0.05).

### 3.6 Enzymatic Haemolymph Parameters

Dietary norbixin exerted significant effects on haemolymph enzyme activities (*p* < 0.05, Table 7). ALT and AST levels decreased progressively, reaching minimum values of 19.23 U/L and 69.55 U/L, respectively, at 0.15 g/kg. Conversely, LDH activity declined sharply from 732.28 U/L at 0.05 g/kg to 679.40 U/L at 0.15 g/kg (*p* < 0.05). In contrast, AKP and ACP activities did not show significant changes across treatments (*p* > 0.05).

**TABLE 7:** Effects of norbixin‐enriched diets on *Macrobrachium nipponense*: assessment of enzymatic haemolymph parameters during a 56‐day feeding trial.

	Norbixin (g/kg)					ANOVA	Linear	Quadratic
Parameters	0.0	0.05	0.10	0.15	0.20	*p*‐value (*F*)	*p*‐value (*R* ^2^)	*p*‐value (*R* ^2^)
Alanine aminotransferase (U/L)	28.16 ± 1.38^a^	24.85 ± 0.93^b^	24.72 ± 0.62^b^	19.23 ± 0.51^d^	21.30 ± 0.53^c^	<0.001 (37.581)	0.020 (0.841)	0.008 (0.984)
Aspartate aminotransferase (U/L)	78.29 ± 1.24^a^	77.36 ± 1.13^b^	72.42 ± 0.91^c^	69.55 ± 0.97^d^	72.35 ± 0.82^c^	<0.001 (37.464)	0.024 (0.848)	0.034 (0.926)
Alkaline phosphatase (U/L)	182.82 ± 1.79	183.99 ± 2.32	182.76 ± 2.21	184.67 ± 2.08	185.83 ± 1.81	0.135 (5.014)	0.082 (0.698)	0.192 (0.852)
Acid phosphatase (U/L)	302.34 ± 2.39	299.9 ± 2.46	299.22 ± 2.12	310.38 ± 1.10	308.13 ± 1.29	0.138 (3.715)	0.079 (0.728)	0.781 (0.223)
Lactate dehydrogenase (U/L)	715.92 ± 5.42^b^	732.28 ± 5.82^a^	709.15 ± 3.70^c^	679.40 ± 2.86^e^	682.93 ± 2.64^d^	0.006 (7.713)	0.011 (0.944)	0.014 (0.869)

*Note*: Values represent mean ± standard deviation (*M* ± *SD*, *n* = 3). Different superscripts in the same raw with values ranging from a to e indicate a significant difference (*p* < 0.05).

### 3.7 Hepatopancreatic Antioxidant Activities

Dietary norbixin supplementation significantly influenced antioxidant parameters in the hepatopancreas (*p* < 0.05, Table 8). T‐AOC increased markedly, peaking at 7.67 U/mg protein in the 0.15 g/kg group. CAT activity declined significantly from 19.05 U/mg protein in the control to 14.12 U/mg protein at 0.15 g/kg. Similarly, SOD levels decreased progressively, with the lowest activity (7.27 U/mg protein) observed at 0.15 g/kg. MDA concentrations were reduced consistently, reaching the minimum value of 8.26 nmol/mg protein at 0.15 g/kg (*p* < 0.05). In contrast, GPx activity remained unaffected (*p* > 0.05).

**TABLE 8:** Effects of norbixin‐enriched diets on *Macrobrachium nipponense*: assessment of hepatopancreatic antioxidant activities during a 56‐day feeding trial.

	Norbixin (g/kg)					ANOVA	Linear	Quadratic
Parameters	0.0	0.05	0.10	0.15	0.20	*p*‐value (*F*)	*p*‐value (*R* ^2^)	*p*‐value (*R* ^2^)
Total antioxidant capacity (U/mg protein)	6.04 ± 0.18^d^	5.73 ± 0.15^e^	6.59 ± 0.19^c^	7.67 ± 0.22^a^	6.89 ± 0.25^b^	<0.001 (37.758)	0.039 (0.872)	0.041 (0.867)
Superoxide dismutase (U/mg protein)	9.62 ± 0.37^a^	8.70 ± 0.32^b^	8.42 ± 0.29^c^	7.27 ± 0.33^e^	8.21 ± 0.20^d^	<0.001 (16.933)	0.013 (0.901)	0.012 (0.923)
Glutathione peroxidase (U/mg protein)	33.13 ± 2.19	33.16 ± 1.88	32.78 ± 1.85	33.83 ± 1.12	32.49 ± 1.23	0.178 (3.981)	0.826 (0.238)	0.128 (0.828)
Catalase (U/mg protein)	19.05 ± 0.62^a^	14.53 ± 0.71^b^	14.48 ± 0.69^b^	14.12 ± 0.38^c^	14.42 ± 0.41^b^	<0.001 (45.763)	0.028 (0.839)	0.033 (0.867)
Malondialdehyde (nmol/mg protein)	11.12 ± 0.56^a^	10.52 ± 0.25^b^	9.23 ± 0.39^c^	8.26 ± 0.42^d^	9.25 ± 0.44^c^	<0.001 (20.568)	0.011 (0.925)	0.039 (0.868)

*Note*: Values represent mean ± standard deviation (*M* ± *SD*, *n* = 3). Different superscripts in the same raw with values ranging from a to e indicate a significant difference (*p* < 0.05).

### 3.8 Digestive Enzyme Activities

Dietary norbixin supplementation significantly enhanced digestive enzyme activities in *M. nipponense* (*p* < 0.05, Table 9). Protease, lipase, cellulase, and amylase activities increased progressively with supplementation, reaching maximum values of 2.11 U/mg protein, 1.32 U/mg protein, 0.40 U/mg protein, and 3.28 U/mg protein, respectively, at 0.15 g/kg (*p* < 0.05).

**TABLE 9:** Effects of norbixin‐enriched diets on *Macrobrachium nipponense*: assessment of digestive enzyme activities during a 56‐day feeding trial.

	Norbixin (g/kg)					ANOVA	Linear	Quadratic
Parameters	0.0	0.05	0.10	0.15	0.20	*p*‐value (*F*)	*p*‐value (*R* ^2^)	*p*‐value (*R* ^2^)
Protease (U/mg protein)	1.85 ± 0.05^d^	1.91 ± 0.07^c^	1.93 ± 0.04^c^	2.11 ± 0.03^a^	2.06 ± 0.04^b^	<0.001 (43.037)	0.019 (0.968)	0.041 (0.860)
Lipase (U/mg protein)	1.06 ± 0.03^d^	1.15 ± 0.04^c^	1.23 ± 0.05^b^	1.32 ± 0.03^a^	1.25 ± 0.03^b^	<0.001 (32.856)	0.011 (0.947)	0.007 (0.921)
Cellulase (U/mg protein)	0.22 ± 0.04^d^	0.27 ± 0.04^c^	0.27 ± 0.05^c^	0.40 ± 0.02^a^	0.35 ± 0.02^b^	<0.001 (39.117)	0.014 (0.921)	0.006 (0.952)
Amylase (U/mg protein)	2.72 ± 0.05^c^	2.78 ± 0.05^c^	2.81 ± 0.04^c^	3.28 ± 0.05^a^	3.15 ± 0.04^b^	<0.001 (45.334)	0.006 (0.915)	0.003 (0.991)

*Note*: Values represent mean ± standard deviation (*M* ± *SD*, *n* = 3). Different superscripts in the same raw with values ranging from a to e indicate a significant difference (*p* < 0.05).

### 3.9 Intestinal Microflora

Dietary norbixin supplementation significantly affected intestinal microbial populations (*p* < 0.05, Table 10). TBC values declined from 8.16 log_10_ CFU/g in the control to 7.15 log_10_ CFU/g at 0.15 g/kg, while LAB counts increased markedly, peaking at 2.04 log_10_ CFU/g in the same group (*p* < 0.05).

**TABLE 10:** Effects of norbixin‐enriched diets on *Macrobrachium nipponense*: assessment of intestinal microflora during a 56‐day feeding trial.

	Norbixin (g/kg)					ANOVA	Linear	Quadratic
Parameters	0.0	0.05	0.10	0.15	0.20	*p*‐value (*F*)	*p*‐value (*R* ^2^)	*p*‐value (*R* ^2^)
Total bacteria count (log_10_ CFU/g)	8.16 ± 0.29^a^	7.92 ± 0.14^b^	7.31 ± 0.18^c^	7.15 ± 0.16^d^	7.39 ± 0.15^c^	<0.001 (17.727)	0.030 (0.827)	0.025 (0.892)
Lactic acid bacteria (log_10_ CFU/g)	1.54 ± 0.04^e^	1.67 ± 0.08^d^	1.75 ± 0.07^c^	2.04 ± 0.04^a^	1.84 ± 0.05^b^	0.028 (4.801)	0.007 (0.954)	0.003 (0.979)

*Note*: Values represent mean ± standard deviation (*M* ± *SD*, *n* = 3). Different superscripts in the same raw with values ranging from a to e indicate a significant difference (*p* < 0.05).

### 3.10 Total Carotenoid Content

Dietary norbixin supplementation markedly increased total carotenoid content in the muscle, shell, and hepatopancreas (*p* < 0.05, Table 11). In muscle tissue, carotenoid concentration rose from 4.74 µg/g in the control group to a peak of 25.12 µg/g at 0.15 g/kg (*p* < 0.05). Shell carotenoid content exhibited a pronounced enhancement, increasing from 7.85 µg/g in the control to 76.33 µg/g at 0.15 g/kg (*p* < 0.05). Similarly, hepatopancreatic carotenoids increased from 5.99 µg/g in the control to 47.19 µg/g at 0.15 g/kg (*p* < 0.05).

**TABLE 11:** Effects of norbixin‐enriched diets on *Macrobrachium nipponense*: assessment of total carotenoid content during a 56‐day feeding trial.

	Norbixin (g/kg)					ANOVA	Linear	Quadratic
Parameters	0.0	0.05	0.10	0.15	0.20	*p*‐value (*F*)	*p*‐value (*R* ^2^)	*p*‐value (*R* ^2^)
Muscle (µg/g)	4.74 ± 0.29^d^	19.12 ± 2.13^c^	24.15 ± 1.10^b^	25.12 ± 0.95^a^	24.26 ± 0.98^b^	<0.001 (74.702)	0.034 (0.971)	0.029 (0.853)
Shell (µg/g)	7.85 ± 0.40^e^	38.61 ± 2.59^d^	66.22 ± 3.75^c^	76.33 ± 3.87^a^	73.11 ± 2.94^b^	<0.001 (45.932)	0.003 (0.982)	0.007 (0.889)
Hepatopancreas (µg/g)	5.99 ± 0.69^e^	23.19 ± 1.89^d^	27.31 ± 1.35^c^	47.19 ± 2.25^a^	38.81 ± 2.01^b^	<0.001 (36.786)	0.029 (0.863)	0.035 (0.918)

*Note*: Values represent mean ± standard deviation (*M* ± *SD*, *n* = 3). Different superscripts in the same raw with values ranging from a to e indicate a significant difference (*p* < 0.05).

### 3.11 Whole‐Body Proximate Composition

Dietary norbixin significantly influenced the whole‐body proximate composition (*p* < 0.05, Table 12). Moisture content decreased from 68.78% in the control to 64.26% at 0.15 g/kg (*p* < 0.05). Crude protein increased from 18.82% in the control to 22.02% at 0.15 g/kg, while crude lipid content rose from 6.73% to 9.96% at the same inclusion level (*p* < 0.05). In contrast, ash content decreased from 4.80% in the control to 3.28% at 0.15 g/kg (*p* < 0.05).

**TABLE 12:** Effects of norbixin‐enriched diets on *Macrobrachium nipponense*: assessment of whole‐body proximate composition during a 56‐day feeding trial.

Parameters			Norbixin (g/kg)			ANOVA	Linear	Quadratic
	0.0	0.05	0.10	0.15	0.20	*p*‐value (*F*)	*p*‐value (*R* ^2^)	*p*‐value (*R* ^2^)
Moisture (%)	68.78 ± 0.44^a^	68.69 ± 0.28^a^	68.77 ± 0.25^a^	64.26 ± 0.39^c^	67.99 ± 0.28^b^	<0.001 (68.764)	0.028 (0.906)	0.031 (0.904)
Crude protein (%)	18.82 ± 0.22^d^	18.86 ± 0.41^d^	18.98 ± 0.43^c^	22.02 ± 0.31^a^	19.41 ± 0.35^b^	<0.001 (54.788)	0.010 (0.905)	0.006 (0.916)
Crude lipid (%)	6.73 ± 0.25^e^	7.83 ± 0.19^d^	8.52 ± 0.15^c^	9.96 ± 0.12^a^	8.83 ± 0.17^b^	<0.001 (49.199)	0.003 (0.947)	0.006 (0.945)
Ash (%)	4.80 ± 0.16^a^	4.73 ± 0.24b	4.54 ± 0.32^c^	3.28 ± 0.21^e^	4.23 ± 0.19^d^	<0.001 (34.349)	0.024 (0.837)	0.035 (0.889)

*Note*: Values represent mean ± standard deviation (*M* ± *SD*, *n* = 3). Different superscripts in the same raw with values ranging from a to e indicate a significant difference (*p* < 0.05).

## 4 Discussion

### 4.1 Water Quality Indicators

Assessment of water quality parameters revealed no significant differences among treatment groups in temperature, pH, ammonium, nitrite, nitrate, phosphate, or total hardness. In contrast, dissolved oxygen exhibited a clear response to norbixin inclusion, with the lowest levels recorded in the control and the highest in groups receiving 0.15 and 0.20 g/kg. This pattern is consistent with earlier findings in *L. vannamei*, where astaxanthin supplementation elevated dissolved oxygen concentrations (Niu et al. 2009). The observed increases in oxygen availability in norbixin‐fed groups emphasise the role of carotenoid pigments in mitigating oxidative stress within aquatic systems. Poor nutrition and unfavourable environmental conditions are known to disrupt metabolic processes and exacerbate free radical production, with immune cells being especially susceptible due to their reliance on membrane receptor signalling. Incorporation of antioxidants such as carotenoids into crustacean diets may counteract these effects by attenuating oxidative damage, enhancing health status, lowering organismal stress, reducing oxygen consumption, and indirectly increasing dissolved oxygen in the aquatic environment (Chien and Shiau 2005; Niu et al. 2009; Elbahnaswy and Elshopakey 2024).

### 4.2 Growth Performance and Feed Efficiency

The present investigation clearly demonstrates that dietary incorporation of norbixin markedly improves growth indices while reducing the FCR in *M. nipponense*. These outcomes align with previous findings across various crustacean species, including *Portunus trituberculatus* (Deng et al. 2024), *L. vannamei* (Shen et al. 2024), *P. clarkii* (Zhang et al. 2023), *Penaeus monodon* (Wang et al., 2021), and *Marsupenaeus japonicus* (Wang et al. 2018), all of which consistently highlight the growth‐enhancing effects of dietary carotenoids. Mechanistically, carotenoids are believed to optimise nutrient utilisation by stimulating digestive enzyme activity, modulating metabolic energy pathways, and promoting intestinal structural integrity (Hertrampf and Piedad‐Pascual, 2012). The reduction in FCR observed here may also be attributable to shortened molting intervals and suppression of oxidative enzymes such as NADPH oxidase, thereby conserving energy for somatic growth (Mao et al., 2017). Additionally, norbixin may facilitate ecdysteroid secretion, a hormone group essential for molting and development, further supporting both the present findings and earlier reports (Liu et al. 2022; Ettefaghdoost and Haghighi 2025).

### 4.3 Metabolic Regulation

Norbixin supplementation induced pronounced alterations in serum biochemical parameters, characterised by reductions in triglycerides, glucose, uric acid, creatinine, and urea, alongside increases in both HDL and LDL cholesterol. Collectively, these changes indicate an enhancement of lipid and energy metabolism. The stability of total cholesterol, calcium, and phosphorus levels likely reflects their tight physiological homeostasis. In contrast, declines in triglyceride and glucose concentrations suggest improved metabolic efficiency and reduced systemic stress (Zhou et al. 2017). These findings are consistent with earlier research emphasising the role of carotenoids in alleviating oxidative stress and modulating lipid metabolism (Ettefaghdoost et al. 2025). The metabolic benefits of norbixin may arise from its antioxidative properties, its ability to attenuate lipid peroxidation pathways (Mahfuzur et al. 2018), and its regulatory influence on lipid transport and biosynthetic enzymes (Ciji and Akhtar 2021). Taken together, these mechanisms support the role of norbixin as a natural dietary modulator of metabolic resilience in aquaculture species.

### 4.4 Immune Modulation

Dietary enrichment with norbixin elicited substantial improvements in immune‐related parameters, evidenced by elevations in TP, ALB, and haemocyte counts (HC, GC, SGC, THC), alongside a decline in serum CORT. These results parallel the immunostimulatory roles previously documented for carotenoids such as astaxanthin in crustaceans (Flores et al. 2007; Chuchird et al. 2015; Wang et al. 2018; Cheng and Wu 2019; Weilong et al. 2019; Eldessouki et al. 2022). Increases in THC are particularly noteworthy, as they signify strengthened innate immune defences. Likewise, higher LYZ and PO activities in norbixin‐fed groups point to augmented immune responsiveness (Zhang et al. 2015). These enzymes constitute critical components of innate immunity, functioning in bacterial clearance and pathogen encapsulation, respectively (Zhi et al. 2018; Ettefaghdoost et al. 2025). Furthermore, the observed reductions in hepatic enzyme markers (LDH, ALT, AST), without significant variation in AKP, suggest improved hepatocellular integrity and reduced liver stress. These hepatoprotective outcomes are consistent with prior studies on *P. clarkii* and *P. monodon* (Chen et al. 2023; Wang et al., 2021; Zhang et al. 2023), reinforcing norbixin's dual functionality as both an immunostimulant and hepatoprotectant.

### 4.5 Antioxidant Defence

Inclusion of norbixin in the diet markedly enhanced T‐AOC, corroborating earlier reports in *Eriocheir sinensis*, *L. vannamei*, and *P. monodon* (Chien et al. 2003; Pan et al. 2003; Jin et al. 2014; Jiang et al. 2020; Fang et al. 2021). Elevated T‐AOC reflects the integrated activity of enzymatic and non‐enzymatic antioxidant pathways, indicating a strengthened systemic defence against oxidative stress. Concurrent reductions in MDA, CAT, and SOD further imply diminished oxidative burden, most likely due to more effective neutralisation of ROS. The decreases in CAT and SOD activities may represent a compensatory response to lower ROS generation, a phenomenon also reported in prior studies (Abele and Puntarulo 2004; Bakiu et al. 2024; Ettefaghdoost et al. 2025). Lower MDA concentrations additionally signify improved membrane stability and reduced lipid peroxidation, underscoring norbixin's protective role against cellular oxidative damage. These results collectively highlight norbixin as a potent dietary antioxidant that safeguards physiological integrity and stress resilience in aquaculture species.

### 4.6 Digestive Enzyme Activity

Supplementation with norbixin significantly enhanced the activity of key digestive enzymes, reflecting improved digestive efficiency and nutrient assimilation. Similar outcomes have been reported in *M. japonicus* and *L. vannamei* (Wang et al. 2018; Fawzy et al., 2022a). The stimulatory effect on enzymatic activity may be linked to norbixin's capacity to stabilise intestinal pH (Rasmussen et al. 2012) and promote the proliferation of beneficial microbial populations such as *Bacillus* and *Lactobacillus* spp. (Tahir et al. 2024; Ettefaghdoost et al. 2025), which themselves produce endogenous digestive enzymes (Saidi et al. 2015; Ettefaghdoost and Haghighi 2025). By fostering a favourable gut microenvironment, norbixin enhances feed breakdown and nutrient uptake, thereby supporting both growth performance and overall health of prawns.

### 4.7 Intestinal Microbiota

Norbixin supplementation induced a marked restructuring of the intestinal microbiota, characterised by reduced TBC and elevated LAB populations, suggesting a shift toward a more favourable microbial community. These outcomes are consistent with prior studies describing the prebiotic‐like effects of carotenoids in aquatic species (Chuchird et al. 2015; Wang et al., 2021; Ettefaghdoost et al. 2025). The selective enrichment of LAB and concurrent suppression of pathogenic bacteria may be attributable to norbixin's influence on gut pH regulation and inter‐microbial competition (Saidi et al. 2015; Sanlier et al. 2024). Such microbial modulation not only supports digestive efficiency but also strengthens immune competence, underscoring norbixin's potential as a multifunctional dietary supplement for sustainable prawn aquaculture.

### 4.8 Carcass Composition

Elevated dietary levels of norbixin resulted in substantial carotenoid accumulation within prawn tissues, accompanied by improvements in carcass quality indicators such as crude protein and lipid content. These observations are consistent with earlier findings (Jiang et al. 2024; Fawzy et al., 2022b; Wade et al., 2017b), confirming both the bioavailability and tissue‐retention capacity of dietary carotenoids. The enhancement in carcass composition may be attributed to norbixin's antioxidative effects (Göçer et al., 2006) and its role in modulating metabolic processes (Wang et al., 2021), which collectively promote protein deposition and lipid storage (Jiang et al. 2024; Ettefaghdoost et al. 2025). Taken together, these results highlight norbixin as a functional dietary additive capable of improving product quality and market value in aquaculture.

## 5 Conclusion

The present study provides strong evidence that dietary supplementation with norbixin is an effective approach to enhance the physiological performance of *M. nipponense*. Inclusion of norbixin at 0.15 g/kg not only improved growth rate, feed efficiency, and survival but also modulated haemato‐biochemical and immune parameters toward a more favourable metabolic state. Elevated T‐AOC, reduced oxidative stress markers, and enhanced digestive enzyme activities collectively demonstrate improved physiological resilience and nutrient utilisation. Furthermore, norbixin supplementation promoted beneficial shifts in gut microbiota and markedly increased carotenoid deposition in muscle, shell, and hepatopancreas, thereby contributing to superior carcass quality. Due to the absence of crustacean‐specific studies evaluating norbixin supplementation, interpretations were contextualised using structurally and functionally related carotenoid compounds. While norbixin differs chemically from classical carotenoids, shared polyene‐based redox properties provide a rational comparative framework until more species‐specific data become available. Importantly, the findings establish norbixin not only as a cost‐effective alternative to high‐value carotenoids such as astaxanthin but also as a strategic feed additive capable of advancing both productivity and profitability in freshwater prawn farming.

## Author Contributions

**Mohammad Ettefaghdoost**: conceptualisation, methodology, formal analysis, data curation, investigation, resources, writing – original draft, writing – review and editing, visualisation, supervision, project administration, funding acquisition. **Hossein Haghighi**: conceptualisation, software, validation, formal analysis, data curation, investigation, writing – original draft. **Adeleh Haghdoost**: investigation, writing – review and editing, validation.

## Funding

The authors have nothing to report.

## Ethics Statement

All methodologies involving animal subjects were executed in accordance with the ethical standards established by the institution and complied with the guidelines stipulated in Directive 2010/63/EU. All pertinent experimental protocols conformed to the recognised principles and regulations governing the management of issues pertaining to experimental animals.

## Conflicts of Interest

The authors declare no conflicts of interest.

## Data Availability

The data that support the findings of this study are available from the corresponding author upon reasonable request.
